# Effects of combined sintilimab and chemotherapy on progression-free survival and overall survival in osteosarcoma patients with metastasis

**DOI:** 10.12669/pjms.40.4.8536

**Published:** 2024

**Authors:** Fan Li, De-bao Zhang, Yue Ma, Yang Song, Xian-liang Duan

**Affiliations:** 1 Fan Li, Department of Orthopaedics, Affiliated Hospital of Beihua University, 132011 Jilin, P. R. China; 2 De-bao Zhang, Department of Orthopaedics, Affiliated Hospital of Beihua University, 132011 Jilin, P. R. China; 3 Yue Ma, Department of Orthopaedics, Affiliated Hospital of Beihua University, 132011 Jilin, P. R. China; 4 Yang Song, Department of Orthopaedics, Affiliated Hospital of Beihua University, 132011 Jilin, P. R. China; 5 Xian-liang Duan, Department of Orthopaedics, Affiliated Hospital of Beihua University, 132011 Jilin, P. R. China

**Keywords:** Metastatic osteosarcoma, Sintilimab, Efficacy, safety

## Abstract

**Objective:**

To explore the safety and efficacy of metastatic osteosarcoma treatment with combined sintilimab injection and chemotherapy.

**Methods:**

We performed a retrospective analysis of 32 patients with metastatic osteosarcoma admitted to the Affiliated Hospital of Beihua University between January 2019 and June 2020. The sample was divided into an observation group, treated with sintilimab injection combined with chemotherapy (n= 16) and a control group, treated with chemotherapy (n = 16). Clinical efficacy and adverse reactions were compared between the two groups.

**Results:**

The overall response rates were 68.75% in the observation group and 31.25% in the control group (*p* < 0.05). The incidences of adverse reactions were 56.25% in the observation group and 81.25% in the control group. This was not a significant difference. In the observation group, the progression-free survival time was 8.13 ± 2.50 months, and the overall survival time was 22.75 ± 4.95 months. These were both significantly longer than the respective 6.44 ± 1.93 months and 19.69 ± 2.68 months in the control group (*p* < 0.05).

**Conclusion:**

The treatment of metastatic osteosarcoma with combined sintilimab injection and chemotherapy was found to prolong progression-free survival and overall survival time without increasing the incidence of adverse reactions.

## INTRODUCTION

In clinical practice, osteosarcoma is the most common primary malignant tumour of bone tissue, primarily affecting adolescents.^1^ It accounts for around 20% of all primary bone tumours.^2^ Most such tumours have metastasised by the time they are diagnosed, mainly to the lungs.^3^ Surgical resection of the primary lesion serves as the main treatment for osteosarcoma, but postoperative recurrence and metastasis are common, resulting in poor prognoses for patients, with low survival rates averaging only five years.^4^

Recent studies have shown satisfactory clinical efficacy in the treatment of metastatic osteosarcoma with certain combinations of chemotherapeutic drugs. Currently, the MAP (high-dose methotrexate, doxorubicin, cisplatin), AP (doxorubicin, cisplatin) and MAID (mesna, doxorubicin, ifosfamide, dacarbazine) are the recommended regimens, among which AP is the most commonly used. The doxorubicin in the AP regimen has a broad anticancer spectrum and satisfactory therapeutic effects on various malignant tumours. Cisplatin is also a commonly used chemotherapy drug in clinical practice with good inhibitory effects on a wide range of malignant tumours.

With the increasing number of studies on immunotherapy for malignant tumours, many immunotherapeutic drugs have now been widely adopted in clinical oncology practice. Sintilimab is an immune-targeted drug independently developed in China and approved for marketing by the China Food and Drug Administration. It has shown satisfactory efficacy with a variety of cancers in clinical trials. In combination with the recommended chemotherapy regimen, it can significantly improve treatment efficacy, safety, and reliability, as well as the immune function and long-term quality of life of patients.^5^,^6^ However, to date, there has been no clinical research on the combination of sintilimab and chemotherapy for the treatment of metastatic osteosarcoma. Therefore, this study explores the efficacy and safety of sintilimab injection combined with chemotherapy in the treatment of metastatic osteosarcoma.

## METHODS

A retrospective analysis was performed using a sample of 32 patients with metastatic osteosarcoma admitted to the Affiliated Hospital of Beihua University between January 2019 and June 2020. This included 18 males and 14 females aged 14–20, with an average age of 16.72 ± 1.61 years. All the patients in our sample had lung metastasis.

### Ethical Approval

The study was conducted in accordance with the tenets of the 2013 revision of the Declaration of Helsinki and approved by the Institutional Ethics Committee of the Affiliated Hospital of Beihua University (No. [2023]025, date: April 18, 2023). Written informed consent was obtained from all participants.

### Inclusion criteria:

- Patients with a diagnosis of primary osteosarcoma (OS) proven by biopsy.
- Patients with a diagnosis of primary OS with organ metastasis proven by a combination of clinical manifestations, lung computed tomography (CT) or systemic positron emission tomography-CT (PET-CT) and histopathology.
- Patients with an estimated survival time ≥6 months.
- Patients and their families voluntarily consented to participate in this study and gave informed consent.

### Exclusion criteria:

- Patients with an estimated survival time of <6 months.
- Patients with central nervous system metastasis.
- Patients with other severe or unstable diseases.
- Patients with liver or kidney dysfunction or bone marrow suppression before treatment.

### Treatment Methods

The patients were divided into an observation group treated with sintilimab injection and the AP regimen (n = 16) and a control group treated with the AP regimen only (n = 16). Both groups received the AP regimen, which consisted of intravenous injection of 25 mg/m² doxorubicin on days 1–3 and 100 mg/m² of cisplatin by intravenous drip for 24 hours on day two, with each cycle lasting 21 days. Both groups of patients were treated for six cycles. The observation group was also administered 200 mg of sintilimab by intravenous drip every three weeks on the first day of chemotherapy.

### Observation Indicators and Determination Criteria

The incidence of adverse reactions in the two groups during treatment was analysed to evaluate the safety of the treatment. To assess clinical efficacy, the progression-free survival (PFS) and overall survival times in the two groups were recorded one month after the completion of the six treatment cycles. Clinical efficacy was classified into four levels per the criteria of the Union for International Cancer Control and the World Health Organisation. These are a complete response (CR), in which all tumour lesions completely disappear for at least one month; a partial response (PR), in which tumour lesions shrink by over 50%, no new lesions occur, and the patient’s condition does not progress for at least one month; disease stable (DS), in which lesions shrink by less than 50% or grow by less than 25% for at least one month; and disease progression (DP), in which tumour lesions grow by more than 25% or new lesions occur. The overall response rate (ORR) = (CR + PR cases) / total cases × 100%. Adverse reactions during treatment were evaluated using the National Cancer Institute Common Toxicity Criteria 3.0 (NCICTC 3.0). Adverse reactions include myelosuppression (decreased red blood cell, white blood cell and platelet counts), nausea and vomiting, fatigue, alopecia and liver and kidney dysfunction.

### Follow-up

All patients were followed up by designated personnel after treatment completion to record PFS and OS times. PFS time refers to the period from the start of treatment to DP. OS time refers to the period from the start of treatment to death or the final patient follow-up.

### Statistical Methods

SPSS 20.0 (IBM Corp., Armonk, NY, USA) software was used for all statistical analyses. Data were expressed as either n (%), with the c^2^ test used for intergroup comparisons; or as mean ± standard deviation (SD; *χ̅*±*S*), with independent sample t-tests used for intergroup comparisons. Differences with *p* < 0.05 were considered statistically significant.

## RESULTS

In the observation group, there was CR in three (18.75%) cases, PR in eight (50%), DS in three (18.75%) and DP in two (12.5%) cases. The adverse reactions seen were a decreased white blood cell count in two (12.5%), anaemia in two (12.5%), a decreased platelet count in three (18.75%) and nausea and vomiting in two (12.5%) cases.

In the control group, CR was seen in one (6.25%), PR in four (25%), DS in seven (43.75%) and DP in four (25%) cases. Adverse reactions in the control group were a decreased white blood cell count in three (18.75%), anaemia in two (12.5%), a decreased platelet count in four (25%), nausea and vomiting in two (12.5%) and fatigue in two (12.5%) cases. The observation group saw a significantly higher ORR than the control group (*p* < 0.05). However, there was no significant difference between the two groups in the incidence of adverse reactions (Table-I). The PFS and OS times in the observation group were significantly higher than those in the control group (*p* < 0.05; Table-II).

**Table-I:** Clinical efficacy and adverse events resulting from the treatment of OS with metastasis with an AP regimen, either alone (control group) or with sintilimab (observation group).

Group	CR	PR	DS	DP	Overall Response (%)	Adverse Reaction (%)
Observation (n = 16), mean ± SD	3	8	3	2	11 (68.75)	9 (56.25)
Control (n = 16), mean ± SD	1	4	7	4	5 (31.25)	13 (81.25)
c^2^ value					4.433	2.327
*p*-value					0.035	0.126

CR: complete response; DP: disease progression; DS: disease stable; PR: partial response; SD: standard deviation.

**Table-II:** Follow-up results of osteosarcoma patients with metastasis treated with an AP regimen alone (control group) or with AP and sintilimab (observation group) (*χ̅*±*S*).

Group	Progression-Free Survival (months)	Overall Survival (months)
Observation (n = 16), mean ± SD	8.13 ± 2.50	22.75 ± 4.95
Control (n = 16), mean ± SD	6.44 ± 1.93	19.69 ± 2.68
*t* value	2.137	2.178
*p*-value	0.041	0.037

SD: standard deviation.

## DISCUSSION

Although the incidence of OS is low, the prognosis of patients is poor because of its high biological malignancy. At clinical diagnosis, this cancer has already metastasised in 10%–20% of patients, with lung metastasis being the most common.^7^ Even with timely treatment after diagnosis, the 5-year survival rate remains very low.^8^,^9^ The primary goal of treating OS, especially when metastatic, is to improve the quality of life and prognosis of the patient. At present, treatment relies mainly on chemotherapy. Both doxorubicin and cisplatin are commonly used broad-spectrum anti-tumour drugs that have good therapeutic effects on various tumours.^10^ These are the drugs used in the first-line AP treatment regimen for OS. However, they have toxic side effects and optimising their clinical applications requires the addition of other drugs to reduce these adverse effects.^11^,^12^ The present study found an ORR in OS patients treated with the AP regimen of 31.25%, which is consistent with the results of previous studies.^13^,^14^

Patients with malignant tumours generally experiences immune dysfunction. In recent years, immunotherapy has emerged as a means of enhancing the body’s immune response to tumours. Xindili is a recombinant human immunoglobulin G4 type programmed cell death protein-1 (PD-1) monoclonal antibody that targets the immune system. It was independently developed in China and forms the active component of sintilimab. Its mechanism of action is to bind to PD-1 receptors, block the PD-1/PD-L1 pathway, relieve the immune suppression of tumour cells in the body, reactivate the anti-tumour activity of T lymphocytes and generate a tumour immune response.^15^,^16^ Research has shown sintilimab to have therapeutic effects in the treatment of various tumour types.^17^ It is, therefore, included in various cancer treatment plans and shows good clinical efficacy.^18^,^19^ This study showed an ORR in the observation group treated with sintilimab and the AP regimen of 68.75%. This was significantly higher than the 31.25% ORR of the control group (*p* < 0.05). Hence, the clinical efficacy of sintilimab combined with the AP regimen in the treatment of metastatic OS is better than that of the AP regimen alone.

As with many chemotherapy drugs, immunotherapy also produces adverse reactions, but the mechanism of their occurrence is currently unclear. Research suggests that these drugs activate the immune response of T cells to cancer cells.^20^ However, the T cells also act on some normal cells, and this excessive autoimmune response leads to adverse reactions to immunotherapy. These adverse reactions are also related to inflammatory factors.^21^ We found no significant difference between the observation group and the control group in the incidence of adverse reactions during the treatment period (*p* > 0.05). However, both the chemotherapy drugs used in the AP regimen are associated with multiple adverse reactions, and the combination of sintilimab with this regimen did not increase the adverse reactions of patients with metastatic OS.

Previous studies have shown that sintilimab can eliminate smiling lesions,^22^ delaying and controlling DP and improving patient survival times and rates. Our results showed significantly higher PFS and OS times in patients treated with sintilimab and the AP regimen than in those treated with the AP regimen alone. Compared with a traditional chemotherapy regimen, sintilimab combined with the AP regimen can prolong the PFS and OS times of patients, effectively improving their prognoses.

### Limitations

However, our study had some shortcomings. Primarily, these were the small sample size and the lack of long-term follow-up. Future research should address these issues in prospective clinical trials to fully validate our results.

## CONCLUSIONS

Sintilimab injection combined with an AP chemotherapy regimen shows high clinical efficacy and safety in the treatment of metastatic OS. It significantly improves the survival time of patients and is worthy of large-scale clinical trials that allow the development of universal guidelines.

### Authors’ Contributions:

**FL** and **XD:** Designed this study, prepared this manuscript, are responsible and accountable for the accuracy and integrity of the work.

**DZ:** Collected and analyzed clinical data.

**YS** and **YM:** Data analysis, significantly revised this manuscript.
